# Crisaborole Loaded Nanoemulsion Based Chitosan Gel: Formulation, Physicochemical Characterization and Wound Healing Studies

**DOI:** 10.3390/gels8050318

**Published:** 2022-05-19

**Authors:** Mohd Nazam Ansari, Gamal A. Soliman, Najeeb Ur Rehman, Md. Khalid Anwer

**Affiliations:** 1 Department of Pharmacology and Toxicology, College of Pharmacy, Prince Sattam Bin Abdulaziz University, Alkharj 11942, Saudi Arabia; g.soliman@psau.edu.sa; 2 Department of Pharmacology, College of Veterinary Medicine, Cairo University, Giza 12211, Egypt; 3 Department of Pharmaceutics, College of Pharmacy, Prince Sattam Bin Abdulaziz University, Alkharj 11942, Saudi Arabia; m.anwer@psau.edu.sa

**Keywords:** nanoemulsion, chitosan, flux, wound healing, anti-inflammatory activity

## Abstract

The development of an effective gel capable of treating eczema remains a challenge in medicine. Because of its greater retention in the affected area, good absorption of wound exudates, and induction of cell growth, nanogel is widely investigated as a topical preparation. Chitosan gel based on nanoemulsions has received much attention for its use in wound healing. In this study, four formulae (CRB-NE1-CRB-NE4) of crisaborole-loaded nanoemulsions (CRB-NEs) were developed using lauroglycol 90 as an oil, Tween-80 as a surfactant, and transcutol-HP (THP) as a co-surfactant. The prepared NEs (CRB-NE1-CRB-NE4) were evaluated for their physicochemical properties. Based on vesicle size (64.5 ± 5.3 nm), polydispersity index (PDI) (0.202 ± 0.06), zeta potential (ZP, −36.3 ± 4.16 mV), refractive index (RI, 1.332 ± 0.03), and percent transmittance (% T, 99.8 ± 0.12) was optimized and further incorporated into chitosan (2%, *w*/*w*) polymeric gels. The CRB-NE1-loaded chitosan gel was then evaluated for its drug content, spreadability, in-vitro release, flux, wound healing, and anti-inflammatory studies. The CRB-NE1-loaded chitosan gel exhibited a flux of 0.211 mg/cm^2^/h, a drug release of 74.45 ± 5.4% CRB released in 24 h with a Korsmeyer-Peppas mechanism release behavior. The CRB-NE1-loaded gel exhibited promising wound healing and anti-inflammatory activities.

## 1. Introduction

The epidermis and dermis layer of skin forms a protective barrier against various harsh environmental attacks such as infection, injuries, and incisions [1]. A wound on the skin is a deterioration of structural and functional integrity of the epithelial tissues, and it can develop due to physical, mechanical, or other influences such as insect/animal bites, accidental injuries, cuts by sharp objects, etc., [2]. This may vary from a minor injury of epithelial tissues of the skin or maybe deeper damage to the subcutaneous tissue and others such as blood vessels, muscles, tendons, bones, parenchymal organs, and nerves [3]. Natural wound healing processes are essential for restoration of disrupted anatomical continuity and functional status of the skin but could be retarded after injuries [4]. Tissue repair and wound healing are a complex process that involves a series of biochemical and cellular reactions, beginning with inflammation and followed by reepithelialization, granulation tissue formation, and remodeling of the extracellular matrix [5]. During the inflammatory phase, macrophages mainly orchestrate the removal of fibrin and fibroblast proliferation. Wound healing proceeds only after the inflammation stops [6]. Although the healing process progresses naturally, an infection can seriously delay this healing process by prolonging the inflammatory phase, disrupting the normal clotting mechanisms, promoting disordered leukocyte function, and ultimately delaying angiogenesis [7].

Currently available methods of wound management including irrigation, debridement, antibiotics, proteolytic enzymes, and tissue grafts are found to be associated with major drawbacks such as invasiveness and are expensive [8]. Therefore, there is need for finding a suitable target for wound healing with anti-inflammatory effects.

Phosphodiesterase 4 (PDE4) isozyme is expressed in almost all inflammatory cells and is a key regulator of inflammatory cytokine production that plays an important role in the regulation of intracellular cAMP levels in these cells and promotes pro-inflammatory responses [9]. PDE4 inhibitor suppresses many inflammatory responses in most inflammatory cells by increasing the cAMP [10]. Therefore, PDE4 inhibitors are the new nonsteroidal, anti-inflammatory agents being investigated for the treatment of skin diseases [9].

**Figure 1:**
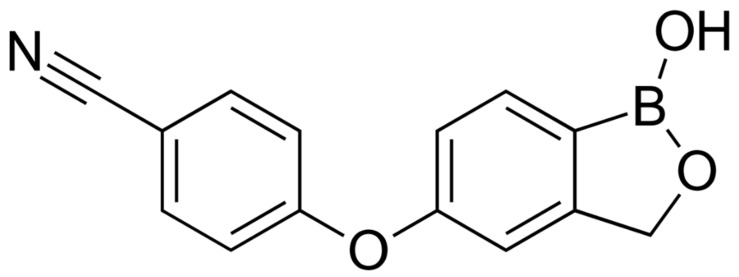
Chemical structure of Crisaborole.

Crisaborole (CRB) is a (5-hydroxy-1,3-dihydro-2,1-benzoxaborole; Figure 1) phosphodiesterase (PDE4) inhibitor that was approved as a drug by the FDA in 2016 and was indicated for the treatment of mild to moderate atopic dermatitis [11,12]. The CRB is marketed as white to off-white ointment (2% *w*/*w*). The animal toxicity studies of crisaborole that were conducted following the systemic and topical administration indicated that crisaborole had a wide safety margin and was available for further application in clinical trials [13].

The therapeutic efficacy of CRB can be improved by incorporation into a suitable nanocarrier. With the advancement in the drug delivery system, nanocarrier-based formulations are being explored for topical delivery. The use of nanoemulsions (NEs) as carrier systems to encapsulate active substances is a unique method of drug delivery. A transparent, thermodynamically stable, isotropic mixture of oil, water, and a surfactant/co-surfactant combination is characterized as NEs [14,15]. After topical administration, lipophilic drugs are frequently localized in the superficial skin layers. Lipophilic drugs incorporated into NEs have been shown to enter the skin in recent studies. NEs can boost the local or systemic distribution of medication as topical carriers [16,17]. The composition and structure of NEs allow them to contain more drug content than other topical formulations such as ointments, creams, gels, and lotions. The finely distributed oil droplet phase of NEs can improve the solubility of weakly water-soluble drugs [18]. As a result of the NEs formulation, the drug will be able to reach the wound’s underlying skin layers, notably the stratum germinativum and dermis, where wound healing and angiogenesis occur, and operate as a microreservoir with a delayed release of the entrapped agent [19]. The goal of this research was to incorporate CRB-loaded NEs into a chitosan polymeric gel to allow for longer drug release and penetration. The new formulation may improve drug absorption deeper into the skin layer, eradicating fungal infections in the dermal region and possibly ending a cyclic recurrence of infection.

## 2. Results and Discussion

### 2.1. Solubility Studies of CRB in Oils, Surfactants and Co-Surfactants

**Figure 2:**
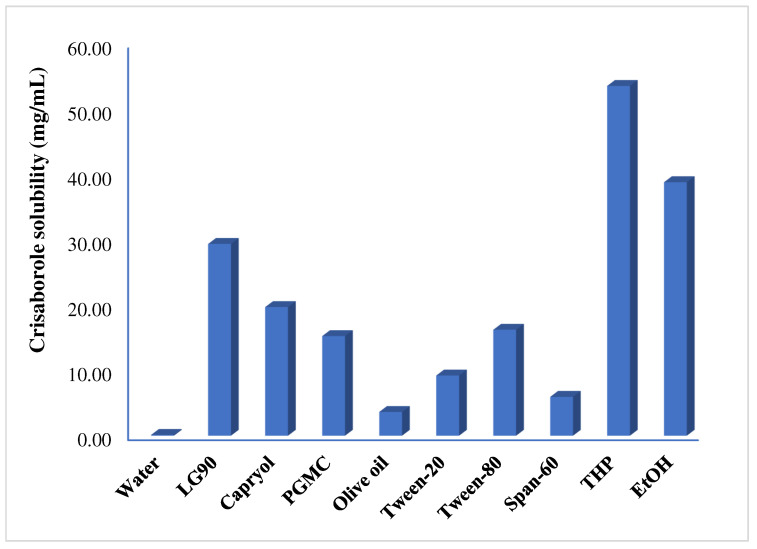
Solubility profile of crisaborole (CRB) in different components.

The solubility of CRB was determined in order to select suitable oil, surfactant, and co-surfactants for the preparation of NEs. The solubility of CRB was presented in Figure 2. Maximum solubility of CRB was determined in LG-90^®^ (oil), Tween-80 (surfactant) and THP (co-surfactant) as 29.31 ± 0.88 mg/mL, 16.19 ± 1.35 mg/mL, and 53.54 ± 2.04 mg/mL, respectively. Based on solubility data, LG-90^®^ as oil, Tween-80 as a surfactant, and THP as co-surfactant were further used for the preparation of a pseudo-ternary phase diagram.

### 2.2. Pseudo-Ternary Phase Diagram

**Figure 3:**
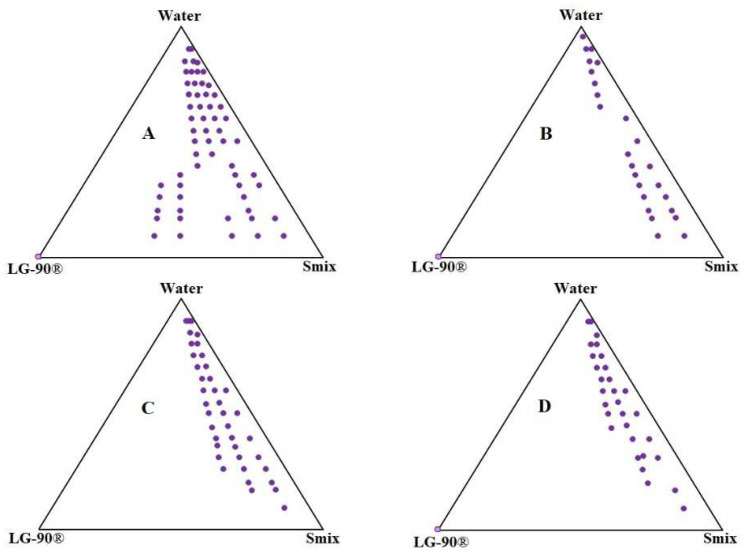
Ternary phase diagrams. (**A**)-1:1; (**B**)-1:2; (**C**)-2:1; (**D**)-1:3.

A Ternary phase diagram was plotted to identify a clear, transparent zone of NEs. Four Smix composed of Tween-80 and THP in the ratio (1:1, 1:2, 2:1, and 3:1) were studied. Large NEs zone in Figure 3A with Smix (1:1) could be seen due to maximum adsorption of the surfactant on the oil/water interface, which results in the lowest droplet size in comparison to Smix, 1:2, 2:1, and 3:1 [20]. This suggests Smix with a 1:1 ratio covers the maximum zone to prepare transparent NEs. However, the lowest NEs zone was found in a Smix 1:2 ratio. From the ternary phase diagram, it was found that the maximum nanoemulsion zone was formed with a Smix ratio of 1:1 (Figure 3B). Therefore, four NEs compositions were selected from a Smix 1:1 ratio, comprising LG-90 (9–45%, *w*/*w*), Smix (10–50%, *w*/*w*) and water (20–80%, *w*/*w*).

### 2.3. Measurement of Physicochemical Properties

The average droplet size, PDI, and ZP of CRB loaded NEs (CRB-NE1-CRB-NE4) were measured in the range from 64.5 ± 5.3 to 123.6 ± 9.8 nm, 0.202 ± 0.06 to 0.303 ± 0.01, and −28.6 ± 4.23 to −36.3 ± 4.16 mV, respectively (Table 1). Among the all developed NEs (CRB-NE1-CRB-NE4), CRB-NE1 had the minimum droplet size (64.5 ± 5.3 nm) with PDI (0.202 ± 0.06) and the highest ZP (−36.3 ± 4.16 mV). The highest ZP values (± ˃ 30 mV) indicate a stable nanoemulsion [21]. The mean values of RI’s for CRB-NE1-CRB-NE4 were measured in the range from 1.302 ± 0.03 to 1.411 ± 0.04. The least RI’s value for CRB-NE1 (1.302 ± 0.03) was measured; it may be the lowest content of oil in the formulation [22,23]. The highest % T was observed for CRB-NE1, indicating a transparent formulation.

### 2.4. Thermodynamic Stability Studies

Thermodynamic studies were conducted to check unstable NEs. Among all the developed NEs (CRB-NE1-CRB-NE4), CRB-NE1 was found to be the most stable NEs on heating and cooling, freeze-thaw cycle, and centrifugation. Therefore, based on physicochemical properties and stability studies, CRB-NE1 was selected for the preparation of CRB-NE1 loaded chitosan gel.

### 2.5. Drug Content Estimation

Drug content analysis revealed that CRB loaded NE1 was uniformly distributed in the gel matrix. The result was 98.68 ± 1.88%, which represented a uniformity of the drug content in the polymer gel matrix. Mixing of the CRB loaded NE1 in the chitosan was observed to be impeccable.

### 2.6. Transmission Electron Microscopy (TEM)

**Figure 4:**
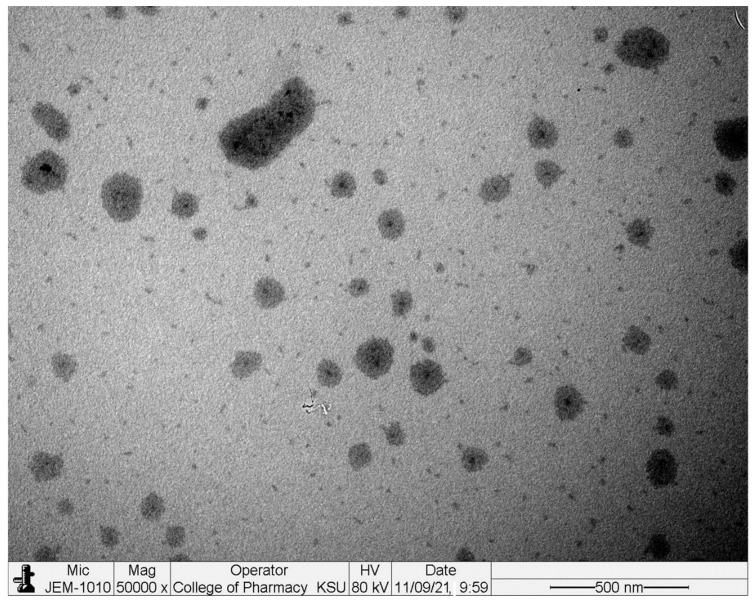
TEM images of optimized NEs (CRB-NE1).

The surface morphology was examined by TEM and exhibited a spherical shape with a smooth surface without any aggregation. Figure 4 clearly indicated that nanodroplets were formed. These experimental data were closely similar to the DLS technique. The nanodroplets appear dark with black surroundings [24].

### 2.7. Spreadability Studies

The spreadability of the CRB gel noted was 7.8 ± 2.1 cm. This parameter plays a significant role in the smearing of the gels on the affected area of the wound. Therapeutic efficacy and topical formulation efficiency depend on the spreadability of the formulation under investigation, which in turn depends on the viscosity of the gel. Patient compliance will be better if the formulation has good spreadability. Thus the CRB gels were found to have the optimum spreadability and, in compliance with the reported value, ranged from 8–9 cm as described by Pal and Chakraborty [25].

### 2.8. pH

The pH of the developed CRB-NE1 loaded chitosan gel was measured as 5 ± 1.6, 5.0 ± 1.1 and 4.9 ± 1.4 at 0 h, 24 h and 48 h, respectively, which was expected to be due to the chitosan and gel composition. The ideal pH of the skin is between 4.5 and 5.5; therefore, the prepared gel could be considered compatible for topical dermal application.

### 2.9. Drug Release and Kinetics Studies

**Figure 5:**
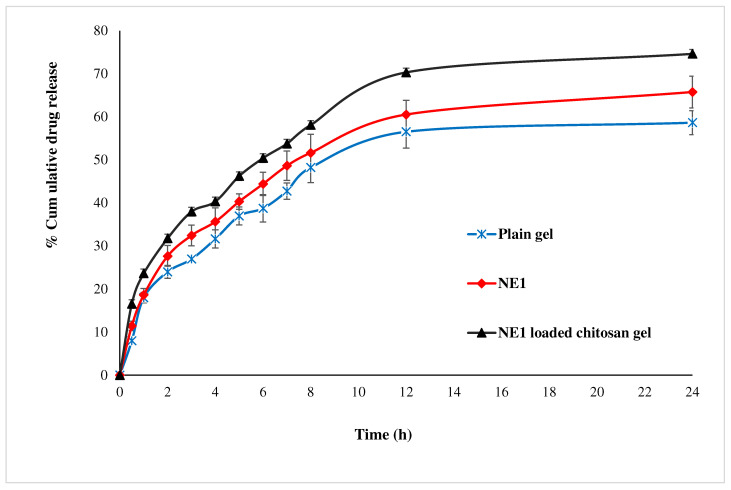
Release profile of optimized NEs (CRB-NE1), the plain gel, and the CRB-NE1-loaded chitosan gel.

In this study, a gelling agent concentration of 2% *w*/*v* chitosan generated a gel with the desired viscosity for topical administration. The cumulative release profile of CRB from optimized NEs (CRB-NE1), the plane gel, and the CRB-NE1-loaded chitosan gel are presented in Figure 5. NEs (CRB-NE1), the plane gel, and the CRB-NE1-loaded chitosan gel enhanced the release of CRB for 24 h. The NEs (CRB-NE1), and the CRB-NE1-loaded chitosan gel exhibited 65.65 ± 3.7% and 74.45 ± 5.4% CRB released in 24 h, respectively. The surfactant/co-surfactants (Tween^®^-80/THP^®^) have been tested for the creation of CRB loaded NEs, allowing for increased CRB release in topically administered dosage forms. As CRB is hydrophobic in nature, a limited release of CRB was thus observed from the plain gel. As a result, it was discovered that the CRB-NE1-loaded chitosan gel successfully increased release of CRB. Furthermore, release data were fitted with different kinetic models to reveal the CRB release mechanism. The coefficient of correlation value of Kormeyer-Peppas (R^2^ = 0.9733) was higher in comparison to zero order (R^2^ = 0.7038), first order (R^2^ = 0.8480), and Higuchi model (R^2^ = 0.9398), indicating a Korsmeyer-Peppas mechanism release behavior [26]. The exponent (*n* = 0.38) of the Korsmeyer-Peppas model indicated a Fickian diffusion release mechanism [27].

### 2.10. Determination of Partition Coefficient, Permeation Coefficient and Flux

The partition coefficient (Ko/w) and permeability coefficient (Kp) of the CRB-NE1-loaded chitosan gel were measured as 2.48 and 0.0516, respectively. The flux of CRB from the CRB-NE1-loaded chitosan gel (0.2112 mg/cm^2^/h) was measured as higher in comparison to the plan gel (0.03038 mg/cm^2^/h), probably due to the nano-sized droplets incorporated in the chitosan gel [28].

### 2.11. Wound Healing Studies

#### 2.11.1. Acute Dermal Toxicity

The CRB-NE1-loaded chitosan gel application was found to be safe in an acute dermal toxicity test. There was no erythema or edema reported after 24 h of application in the shaved area. When rats were monitored for 14 days after the application of the gel, no mortality or signs of toxicity were observed.

#### 2.11.2. Evaluation of Wound Healing Activity

##### Excision Wound Model

**Figure 6:**
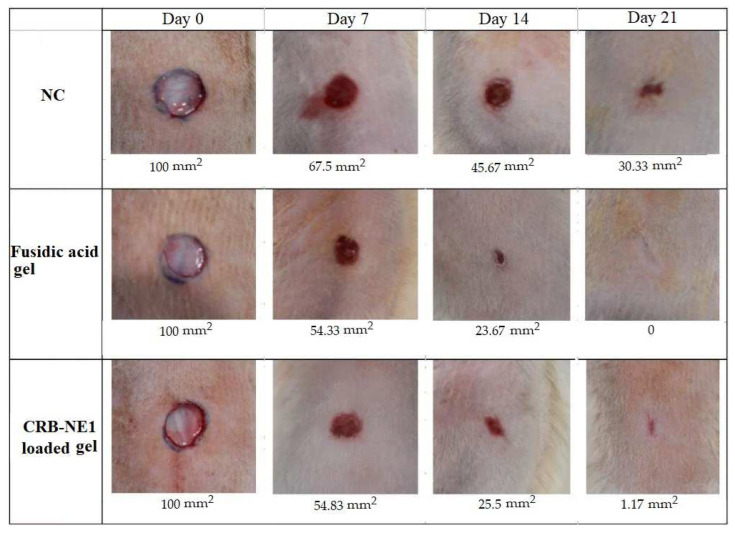
Photographs of wound healing and wound size (mm^2^) at different time intervals in excision wound model in rats.

In the excision wound rat’s model, the sizes of wounds were observed on the 7, 14 and 21 post-wounding days, as shown in Figure 6. Topical application of the reference fusidic acid gel (2%) and the CRB-NE1-loaded chitosan gel on the 7, 14 and 21 post-wounding days reduced wound sizes of rats in comparison to negative control group (Figure 6). On these days, the wound sizes in the reference fusidic acid gel and the CRB-NE1-loaded chitosan gel groups were almost similar and there was no considerable difference between both groups.

**Figure 7:**
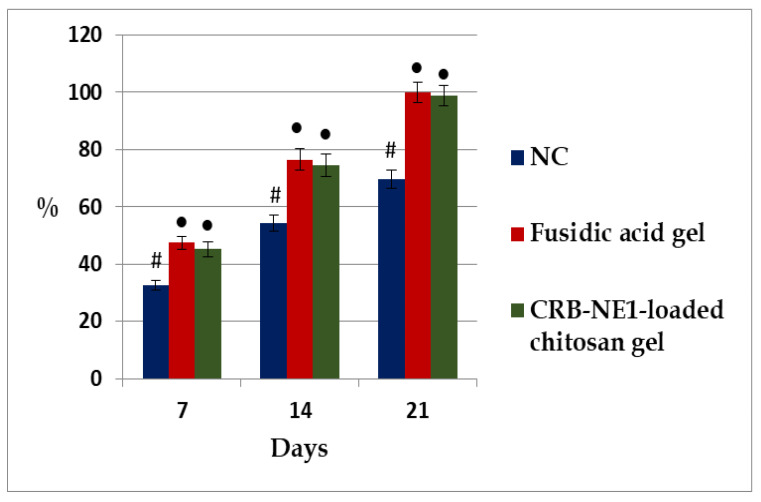
Percentages of wound contractions at different time intervals in excision wound model in rats. Values are expressed as mean ±S.E.M., *n* = 6 rats/group. ● indicates significance compared to NC group at *p* < 0.05. # indicates significance compared to fusidic acid gel group at *p* < 0.05.

In addition, the percentages of wound contraction were calculated on the 7, 14 and 21 post-wounding days (Figure 7). The epithelization involves the migration and proliferation of the newly formed epithelial cells towards damaged wound beds [29]. In the present study as well, topical treatment of the wounds with the reference fusidic acid gel depicted a significant increase in the percentage of wound contraction when compared to the negative control group. A promising significant effect was also observed for the group treated with the CRB-NE1-loaded chitosan gel (Figure 7). Interestingly, the effect of the CRB-NE1-loaded chitosan gel is seen to be comparable to that of the reference fusidic acid gel. These findings were confirmed by the epithelialization periods results (Table 2). The time required for complete epithelialization of the excision wound is an important parameter to assess the wound healing process. The group treated with 2% base gel took a longer time (28.53 ± 1.75 days) to achieve complete epithelialization. Complete epithelialization was observed on the 20.72 ± 1.18 post-wounding day in the fusidic acid gel-treated group, whereas the CRB-NE1-loaded chitosan gel demonstrated similar effect on the 21.45 ± 1.46 post-wounding day. A shorter epithelization period might be due to the adequate viability of epithelial cells [30]. It is interesting that the epithelialization period in the CRB-NE1-loaded chitosan gel-treated group was not significant and comparable with that observed in the reference fusidic acid gel-treated group. The CRB-NE1-loaded chitosan gel showed the same wound healing effect as the standard fusidic acid gel. The CRB-NE1-loaded chitosan gel formulation can thus be promising for topical application, especially for healing wounds in the same way as standard fusidic acid gel. Further experimental and clinical studies are required in the future to confirm the results of the present study.

##### Microscopic Evaluation of the Wound

**Figure 8:**
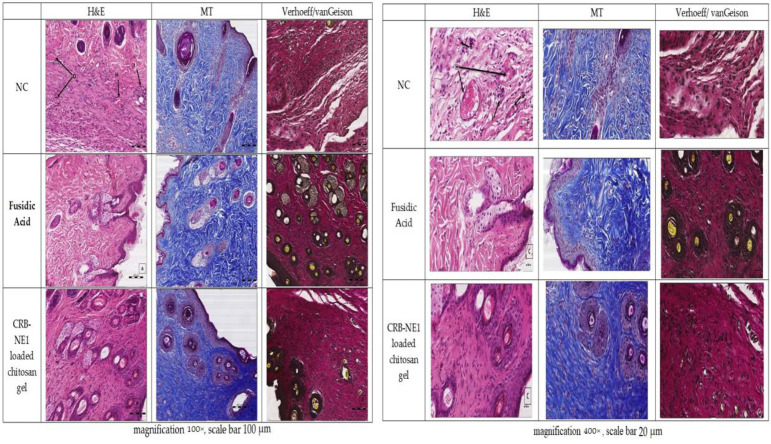
Photomicrographs of skin samples of 21 days after treatment with CRB-NE1 loaded chitosan gel and fusidic acid gel with hematoxylin and eosin (H&E) stain, Masson Trichrome (MT) stain, and Verhoeff/vanGeison stain (magnification 100× and 400×). Abbreviations indicate Hyperemia (H), presence of red blood cells within tissue outside blood vessels which indicate hemorrhage (R), Occlusion of blood vessels with hyaline material (O), and infiltration of tissue by inflammatory cells (I).

To explore the histological effect of the material under investigation (CRB-NE1 loaded chitosan gel) on wound healing compared to both the control group and the acid treated group, three stains (H&E, Masson trichrome, and Verhoef’s hematoxylin counterstained with vanGeison) on formalin fixed paraffin wax embedded representative tissue samples were used. Magnification of photomicrographs A were 100×, and the scale bar was 100 μm in length (Figure 8), while photomicrographs at magnification were 400×, and the scale bars were 20 μm in length (Figure 8). A large size version of these two photomicrographs was mentioned in the Supplementary File (Figures S1–S9).

Using H&E as shown in the two photomicrographs (Figure 8) of the control group revealed suffering of the skin tissue indicated by several pathological events, such as hyperemia (H), the presence of red blood cells within the tissue outside blood vessels which indicated hemorrhage (R), infiltration of the tissue by inflammatory cells (I), and occlusion of blood vessels (O). On the other hand, the two photomicrographs of the fusidic acid group showed a high degree of improvement. Finally, the two photomicrographs of the CRB-NE1 loaded chitosan gel treated group showed a high degree of improvement and almost normal tissue.

Upon using Masson trichrome on the control group, the tissue section showed a decrease in the amount of collagen fibers indicated by a decrease in the blue color. By contrast, the acid gel treated group showed a high degree of improvement by retaining the amount and distribution of collagen fibers. Finally, the CRB-NE1 loaded chitosan gel treated group showed a high degree of improvement in the amount and distribution of collagen fibers (blue color).

Furthermore, upon using Verhoef’s hematoxylin stain counter stained with vanGeison for elastic fibers, we found that the two photomicrographs of the control group showed a decrease of skin elasticity due to a decrease in the amount of elastic fibers, indicated by a decrease in the distribution of the black color of the Verhoef’s hematoxylin stain. On the other hand, the two photomicrographs of the fusidic acid gel treated group showed a high degree of improvement and a retention of the elasticity of the skin. Finally, the two photomicrographs of the CRB-NE1 loaded chitosan gel treated group showed a high degree of improvement and a retention of the elasticity of the skin.

The present study corroborates a previous study reported by Zhang et al. [31]. They used Masson trichrome and found increased mature collagen fibers by increased improvement of tissue due to healing, which led to increased intensity of the blue color of collagen in the tissue sample treated with fusidic acid in their study. Furthermore, the area of the wound healing in this study showed a decrease in elastic fiber, which agrees with the results of Amadeu et al. who compared normal scar versus normal skin in regard to elastin components [32].

### 2.12. Evaluation of Anti-Inflammatory

**Figure 9:**
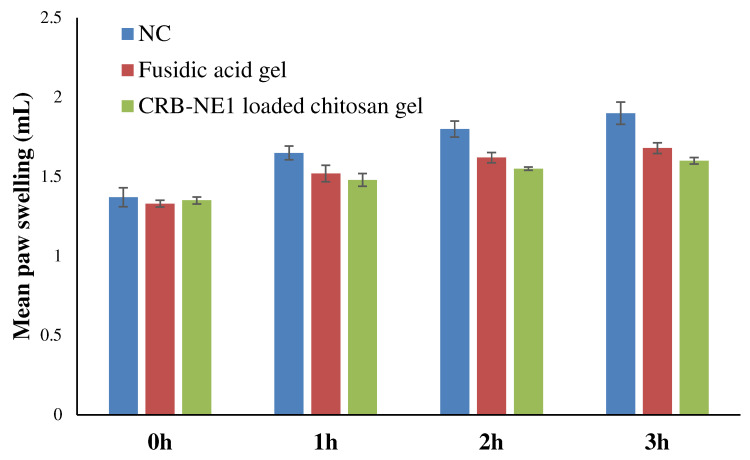
Comparative mean paw swelling by fusidic acid gel and CRB-NE1 loaded chitosan. gel on carrageenan-induced paw edema.

The effect of fusidic acid gel and the CRB-CRB-NE1-loaded chitosan gel formulation on mean paw volume and percentage inhibition of carrageenan-induced edema in rats is shown in Figure 9 and Table 3. Earlier it has been reported that compounds with an anti-inflammatory effect can have wound healing potential also [33]. In the present study, the CRB-CRB-NE1-loaded chitosan gel inhibited edema formation by 51.66% compared to the control group, while the fusidic gel reduced swelling by 35.18%. Based on these findings, it can be concluded that the CRB-NE1-loaded chitosan gel had a 1.5-fold greater anti-inflammatory effect than the fusidic gel. The strongest anti-inflammatory benefits of the CRB-NE1-loaded chitosan gel may be attributable to the highest concentration of CRB accessible at the target side, as well as the nanogel’s ability to enhance drug absorption through the skin.

## 3. Conclusions

In the present study, four NEs (CRB-NE1-CRB-NE4) were developed; and on the basis of physicochemical properties and stability studies, CRB-NE1 was selected for the preparation of the CRB-NE1 loaded chitosan gel. The CRB-NE-loaded chitosan gel showed the significant anti-inflammatory and wound healing activities with a high rate of wound contraction and epithelization. Therefore, this study may be an innovative approach for efficient anti-inflammatory and wound healing activity using CRB-NE-loaded chitosan gel topical application, although further research is necessary to clarify the mechanism involved.

## 4. Materials and Methods

### 4.1. Materials

CRB was purchased from “Beijing Mesochem Technology Beijing, China.” LauroglycolTM-90, Capryol^®^-PGMC, and Transcutol^®^-HP were obtained as a gift sample from Gattefosse (Lyon, France). Tween^®^-80 and Ethanol were procured from “Sigma Aldrich, USA”.

### 4.2. Solubility Studies of CRB in Oils, Surfactants and Co-Surfactants

The solubility of CRB was measured in different components (oils, surfactants, and co-surfactants) in order to develop NEs. The solubility of CRB in different oils (LG-90^®^, PGMC, Capryol^®^, and Olive oil), surfactants (Tween^®^-20, Tween^®^-80, Span^®^-60, and Kollidone^®^-EL), co-surfactants (THP and ethanol) were studied [34]. Briefly, an excess amount of CRB was added in 1 mL of respective solvent (oils/surfactant/co-surfactant) in a clean glass vial. The vials were tightly capped and shaken on a biological shaker (LabTech, LBS-030S, Kyonggi, Korea) for 3 days at 37 ± 1 °C, 100 rpm. After equilibration, samples were centrifuged at 6000 rpm for 10 min, and the supernatant was filtered and diluted suitably, and analyzed by UV/Vis spectroscopy at 250 nm [35].

### 4.3. Pseudo-Ternary Phase Diagram

Based on solubility studies, distilled water (aqueous phase), LG-90^®^ (oil), Tween-80 (surfactant), and THP (co-surfactant) were used for the construction of the ternary phase diagram. The surfactant and co-surfactant (Smix) at different weight ratios (1:1, 1:2, 2:1, and 3:1) were used for the preparation of the triple phase diagram. Oil and Smix were thoroughly mixed at ratio 1:9 to 9:1 and titrated with distilled water till the formation of hazy dispersion [36].

### 4.4. Preparation of Nanoemulsion

Crisaborole (CRB) loaded nanoemulsion (NE) was prepared by the low energy method [24]. Lauroglycol-90^®^ (LG-90^®^) as oil phase, Tween-80^®^ as surfactant, transcutol-HP^®^ (THP) as a co-surfactant, and purified water as aqueous phase were used (Table 4).

### 4.5. Measurement Physicochemical Properties

Droplet size and PDI of the CRB-loaded NEs were measured using a dynamic light scattering (DLS) method using “Malvern zetasizer (ZEN-3600, Malvern Instruments Ltd., Westborough, MA, USA)”. NEs (3.0 mL) were transferred into a plastic cuvette and droplet size and PDI were determined at 25 °C. The same procedure was followed for ZP measurement, except a glass electrode was used in place of the plastic cuvette. The refractive index (RI) values of developed NEs were measured using Abbe’s Refractometer “(Precision Testing Instruments Laboratory, Pfingstweide, Friedberg (Hessen), Germany)” at room temperature [22]. The % transmittance (% T) was measured at 550 nm using a UV/Vis spectrophotometer “(UV-Visible spectrophotometer, Jasco 645, Hachioji, Tokyo, Japan)” [23].

### 4.6. Thermodynamic Stability Studies

The developed NEs (CRB-NE1-CRB-NE4) were subjected to thermodynamic stability to remove the unstable NEs. Stability testing was done by heating and cooling (cycles between 25 and 45 °C), a freeze-thaw cycle (three cycles between −25 °C and 25 °C), and centrifugation [34]. The stability of NEs was analyzed visually for precipitation, phase separation, coalescence, and conversion of nanoemulsion into an emulsion.

### 4.7. Transmission Electron Microscopy (TEM)

The surface morphology of the optimized NEs (CRB-NE1) was examined by using a JEM-1010 transmission electron microscope (JEOL, Tokyo, Japan) operated at 200 kV. A drop of NEs was placed over the grid and stained with phosphotungstic acid. The grid was air-dried, placed on the TEM, and the image was viewed [37].

### 4.8. Preparation of Nanoemulsion Loaded Chitosan Gel

**Figure 10:**
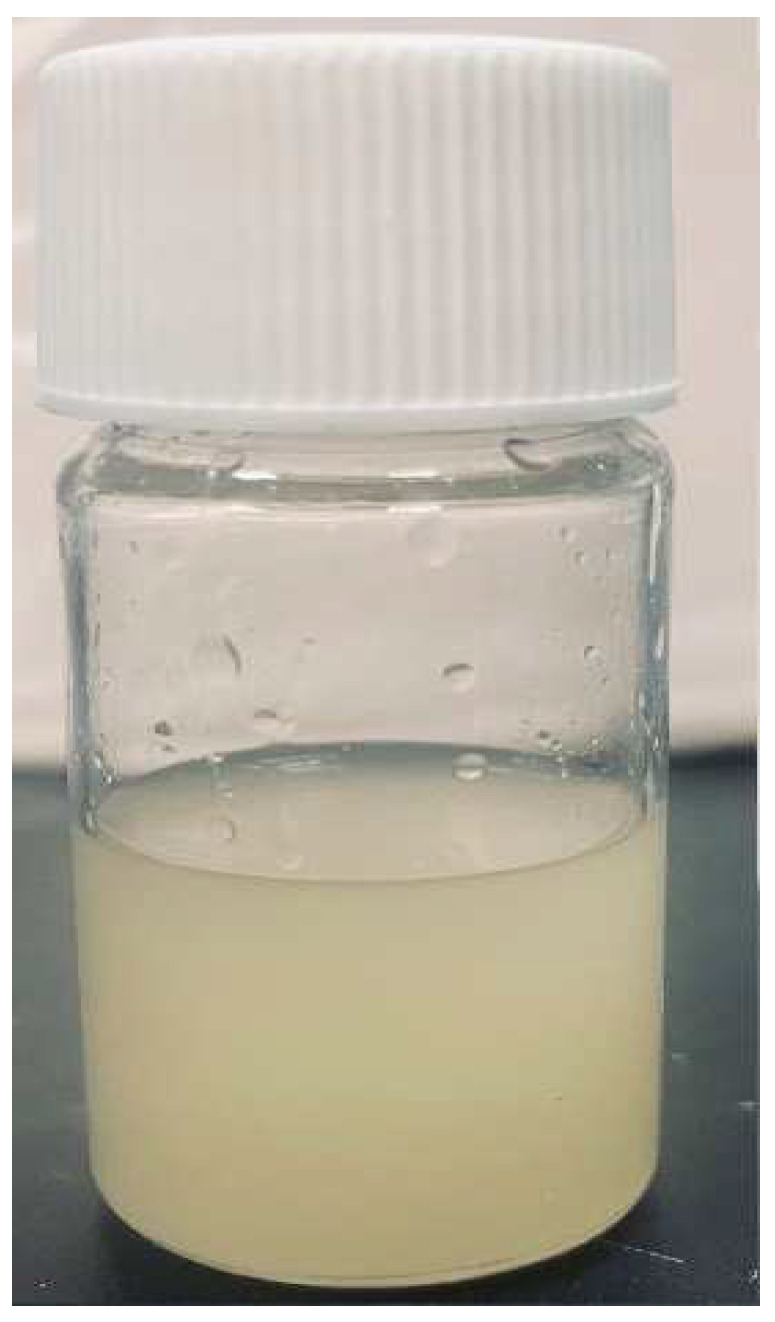
Developed CRB-NE1 loaded chitosan gels.

The prepared CRB-NE1 was incorporated into chitosan gel (2% *w*/*w*). Chitosan gel was prepared by adding in 1% acetic acid, and then dehydrated for 24 h at room temperature to get the desired consistency, and then 0.050% of cetyl pyridinium chloride was added as a preservative. The CRB-loaded nanoemulsion (CRB-NE1) was incorporated into a prepared chitosan gel by continuous stirring with a glass rod to get a smooth and transparent CRB gel (2% *w*/*w*) (Figure 10) [38].

### 4.9. Drug Content Estimation

For drug content, 0.5 g of CRB gel (equivalent of 10 mg of CRB) was dissolved into the 50 mL of methanol, and a set of three samples were then mechanically agitated at 25 °C for 24 h. Thereafter it was centrifuged at 4500 rpm, filtered, diluted appropriately then analyzed using UV spectrophotometric technique (UV-Visible spectrophotometer, Jasco 645, Hachioji, Tokyo, Japan) at wavelength 250 nm [35,39].

### 4.10. Spreadability Studies

A spreadability test was performed by placing CRB gel (0.5 gm) in between two plexiglass plates, followed by tapping with a 500 g weight on the top plate. After a 5 min time interval, the top weight was removed and the gel spread circle diameter was measured [40].

### 4.11. pH

The CRB-loaded NE-based chitosan gel was tested for hydrogen potential using a pH meter (Edge pH series, Hanna instruments, Smithfield, Rhode Island) maintained at 25 °C [41]. The gel (1 gm) was dispersed into distilled water and dipped with a pH electrode, and the reading was then noted (*n* = 3).

### 4.12. Drug Release and Kinetics Studies

In-vitro diffusion study was performed in the modified franz diffusion cell [42], and 50 mg equivalent weight of the gel was placed on the cellophane membrane enclosed with the donor compartment. The acceptor chamber was filled with a phosphate buffer (pH 7.4). The magnetic bead was placed inside the acceptor bottle, and the whole assembly was kept on the magnetic stirrer with a heat adjustment of 37 ± 2 °C at 50 rpm rotations. At pre-determined time intervals, a sample (1 mL) was withdrawn and a sink condition was maintained with a fresh buffer. The aliquot was pre-filtered and analyzed spectrophotometrically at 250 nm (UV-Visible spectrophotometer, Jasco 645, Hachioji, Tokyo, Japan). All experiment design was the same for the set of three samples. Further, data from release studies were fitted to the four kinetic models, namely zero order, first order, Higuchi and Korsmeyer-Peppas models, and the regression analysis was performed using the following equations [43].

$$\begin{array}{ccc} Q_{t}= & Q_{0}+ & k_{0}t \end{array}\;(\mathrm{Zero}\;\mathrm{order})$$

$$\begin{array}{ccc} {\mathrm{logQ}}_{t}= & {\mathrm{logQ}}^{0}- & k_{1}t \end{array}\;/2.303\;(\mathrm{First}\;\mathrm{order})$$

$$Q_{t}={k_{H}t}^{0.5}\;(\mathrm{Higuchi}\;\mathrm{equation})$$

$$M_{t}/M\infty ={\mathrm{kt}}^{n}\;(\mathrm{Korsmaeyer}\text{-}\mathrm{Peppas})$$

### 4.13. Determination of Partition Coefficient, Permeation Coefficient and Flux

Aqueous (distilled water) and organic phase (octanol) were mixed in the 1:1 ratio, shaken, and kept aside for saturation. The plane gel (drug suspended gel) and the CRB-NE1-loaded chitosan gel (equivalent to 10 mg) were dispersed in a separating funnel containing octanol and the mixture, then shaken vigorously and kept aside for 24 h at room temperature. Both octanol and water got separated, then filtered, and analyzed the drug content in both solvents [34]. The partition coefficient was estimated using the equation:

Ko/w = CRB conc. in octanol/CRB conc. in water

Further, the permeability coefficient (Kp) was estimated using the relationship between Ko/w and the molecular weight of the CRB drug (MW 251.05). The Kp was calculated as per the previously reported method [44].

$$\begin{array}{cc} \mathrm{Log}\;\mathrm{Kp}\;\left(\mathrm{cm}/h\right) & \;=-2.72+0.71\;\mathrm{LogK}o/w-0.0061\times \left(\mathrm{MW}\right)\mathrm{LogKp}\;\left(\mathrm{cm}/h\right)\\ & =-2.72+0.71\;\mathrm{LogK}o/w-0.0061\times \left(\mathrm{MW}\right) \end{array}$$

The flux of CRB was calculated using the equation [45].

Flux=LogKp×(Aqueous solubility of CRB)

### 4.14. Wound Healing Studies

#### Animals

Healthy, Wistar albino rats (180–200 g) of either sex were procured from the animal care unit of the College of Pharmacy, Prince Sattam Bin Abdulaziz University (PSAU), Saudi Arabia. The animals were housed in transparent cages under standard laboratory conditions of 12/12 h light dark cycle, at a temperature of 22 ± 2 °C, and provided with a pellet diet and water ad libitum. Before beginning the experiment, all animals were given a week to acclimate to the laboratory conditions. The ethical committee approved the animal study, which was carried out at the College of Pharmacy, PSAU, Saudi Arabia, in accordance with the science and ethical principles for animal care (BERC-00105-19).

### 4.15. Acute Dermal Toxicity

The acute dermal toxicity test was carried out following OECD guideline number 434 [46]. A total of ten female Wistar rats (8–12 weeks) with normal skin texture were chosen, and approximately 10% of the body surface area was shaved from the dorsal area of the trunk 24 h before the study. They were divided into two groups (treatment and control). The CRB-NE1-loaded chitosan gel was applied uniformly to the test site and covered with a porous gauze dressing and non-irritating tape throughout a 24 h exposure period. First, a sighting study was conducted using the 10% gel to determine the starting dose. All animals were examined for signs of erythema and edema, and responses were recorded at 60 min, 24, 48, and 72 h. After 24 h, there was no death or skin irritation, so four more rats from each group were used, with the same dose of gel applied. The animals were observed for the development of any adverse skin reactions immediately after dosing, at least once during the first 30 min, periodically during the first 24 h, with special attention given during the first 4 h, and daily thereafter for a total of 14 days, unless they were found dead.

### 4.16. Evaluation of Wound Healing Activity

#### Excision Wound Model

For the excision model, three groups of six rats each were used. Animals in group I (NC) were given a 2% Base gel topically as a negative control. Groups II (positive control) and III (treated) received the Fusidic acid gel and 2% *w*/*w* the CRB-loaded NE-based chitosan gel, respectively.

**Figure 11:**
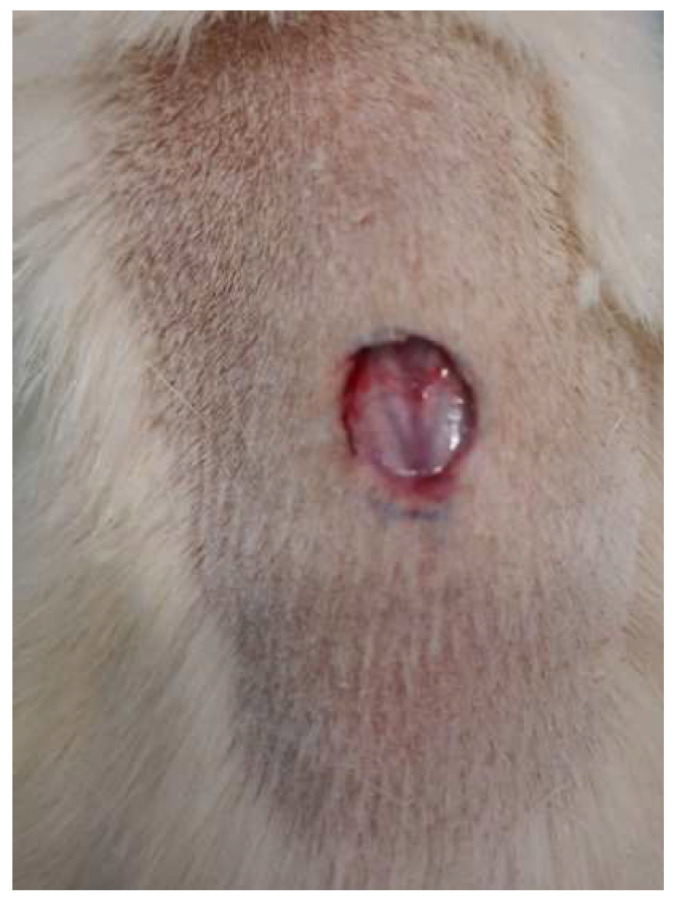
Excision wound on day 0.

The rats were anesthetized with chloral hydrate (400 mg/kg, IP) [47]. The dorsal furs were shaved with a shaving machine prior to the wound area preparation. Using a permanent marker, a 100 mm^2^ circular mark was created, and the full thickness of this circular mark was excised with sterilized forceps and scissors to create the wound (Figure 11). This was considered as day 0. Starting from day 1, the rats were treated with the base, the CRB-loaded NE-based chitosan gel, and the fusidic acid gel as described for the evaluation of wound healing activity. All of the preparations were applied to the wound area daily until the wounds in the test groups healed completely. Every 7 days, the wound area was measured with a transparent sheet and a permanent marker. The transparent sheet was traced out on 1 mm^2^ scale graph paper. The period of epithelialization and the percentage of wound contraction were used to evaluate wound healing activities [48,49]. The percentage of wound contraction was calculated as follows:

(1) $$\%\;\mathrm{Wound}\;\mathrm{Contraction}=\frac{\mathrm{Wound}\;\mathrm{Area}\;\mathrm{on}\;\mathrm{day}\;0-\mathrm{Wound}\;\mathrm{Area}\;\mathrm{on}\;\mathrm{day}\;n}{\mathrm{Wound}\;\mathrm{Area}\;\mathrm{on}\;\mathrm{day}\;0}\times 100$$

where *n* = the days when the measurement was taken.

### 4.17. Microscopic Evaluation of the Wound

Skin samples for the histopathological study were collected after 21 days of the wound creation from all the groups. The animals were euthanized humanly by chloral hydrate (400 mg/kg, IP), and full-thickness skin samples from the wound area were taken and fixed in formalin solution. The samples were processed in an automatic tissue processor (ASP300s, Leica Biosystems, Richmond, IL, USA). After complete tissue processing, these tissue samples were embedded in blocks of paraffin wax and a 5 µm thickness section was prepared from each tissue block by using a rotary microtome (SHUR/Cut 4500, TBS, Sanford, NC, USA). Each sample was cut into three sections for staining. These stains were H&E for the general histological picture [50], Masson trichrome for connective tissue fibers, and Verhoef hematoxylin for elastic fibers [51,52]. Wound changes were observed under a light microscope by a histopathologist who pathologically assessed photomicrographs.

### 4.18. In Vivo Anti-Inflammatory Activity

The anti-inflammatory activity of the CRB-NE1-loaded chitosan gel was tested using the carrageenan-induced rat hind paw edema method [53,54]. The rats in each group were selected so that the average body weight among the groups was as close as possible. For the evaluation of anti-inflammatory activity, overnight fasted rats (*n* = 18) were randomly divided into 3 groups of six rats each. Rats of group 1 (NC) received only the 2% base gel without drug topically on the plantar surface of the left hind paw by gently rubbing 50 times with the index finger. Group 2 (treated group) and group 3 (positive control group) received the CRB-NE1-loaded chitosan gel and the FA gel, respectively, topically on the plantar surface of the left hind paw by the same mode of application as a base gel. After 3 h, the treated paw was injected subplantarly with 0.1 mL of a 1% carrageenan solution (in normal saline). Using a Plethysmometer (UGO Basile, Gemonio VA, Italy), the paw edema was measured immediately (0 h) and then again after 3 h of carrageenan injection. The following equation was used to calculate the percent swelling of the paw:

$$\%\;Swelling=\frac{V-Vi}{Vi}\times 100$$

where *V* represents the paw volume after 3 h of carrageenan injection and *Vi* represents the initial paw volume.

The average paw swelling in the drug-treated rats was compared to that in the control rats, and the anti-inflammatory effect of the treatments was calculated as the percent inhibition of edema using the following formula:

$$\%\;Paw\;Edema\;Inhibition=\frac{\%\;Swelling\;of\;control\;group-\%\;Swelling\;of\;treated\;group}{\%\;Swelling\;of\;control\;group}\times 100$$

### 4.19. Statistical Analysis

All the results were expressed as mean ±standard error of the mean (SEM). Statistical analysis of the results was carried out by one-way analysis of variance (ANOVA) followed by Bonferroni test. At a 95% confidence level (CI) and *p*-value of 0.05, the results were considered statistically significant. The GraphPad program was used for all data processing (Graph Pad, San Diego, CA, USA).

## Figures and Tables

**Table 1:** Physicochemical properties of developed CRB loaded NEs (CRB-NE1-CRB-NE4).

NEs	Droplet Size (nm)	PDI	ZP (mV)	RI	% T
CRB-NE1	64.5 ± 5.3	0.202 ± 0.06	−36.3 ± 4.16	1.332 ± 0.03	99.8 ± 0.12
CRB-NE2	102.7 ± 8.1	0.273 ± 0.05	−28.6 ± 4.23	1.334 ± 0.05	97.6 ± 0.16
CRB-NE3	85.4 ± 7.1	0.303 ± 0.01	−35.6 ± 3.42	1.364 ± 0.06	98.4 ± 0.14
CRB-NE4	123.6 ± 9.8	0.295 ± 0.02	−34.2 ± 3.54	1.311 ± 0.04	96.5 ± 0.08

**Table 2:** Effect of the reference fusidic acid gel and the CRB-NE1-loaded chitosan gel on the epithelialization periods in rat’s wounds.

Treatments	Epithelialization Period (Days)
NC	28.53 ± 1.75 #
Fusidic acid gel	20.72 ± 1.18 ●
CRB-NE1-loaded chitosan gel	21.45 ± 1.46 ●

Values are expressed as mean ± S.E.M., *n* = 6 rats/group. ● indicates significance compared to NC group at *p* < 0.05. # indicates significance compared to fusidic acid gel group at *p* < 0.05.

**Table 3:** Anti-inflammatory activity of the CRB-NE1-loaded chitosan gel on carrageenan-induced paw edema.

Groups	Percent Swelling	Percent Inhibition
NC	39.68 ± 1.24 #	-
Fusidic Acid gel	25.72 ± 2.01 ●	35.18
CRB-NE1 loaded chitosan gel	19.18 ± 0.64 ●#	51.66

Values are expressed as mean ± S.E.M., *n* = 6 rats/group. ● indicates significance compared to NC group at *p* < 0.05. # indicates significance compared to fusidic acid gel group at *p* < 0.05.

**Table 4:** Composition of CRB loaded NEs.

NEs Codes	Compositions (% *w*/*w*)				Smix
	LG-90^®^	Tween^®^-80	THP^®^	Water	
CRB-NE1	10	40	40	20	1:1
CRB-NE2	12	20	20	48	1:1
CRB-NE3	16	24	24	36	1:1
CRB-NE4	40	10	10	40	1:1

Each mL of NEs contains 100 mg of loaded CRB. **^®^** The registered trademark symbol.

## Data Availability

The data presented in this study are available on request from the corresponding author.

## Supplementary Materials

The following supporting information can be downloaded at: https://www.mdpi.com/article/10.3390/gels8050318/s1, Figure S1: These photomicrographs of control group indicate suffering of skin tissue at both levels of dermis and epidermis indicated by several pathological events such as Hyperemia (H), presence of red blood cells within tissue outside blood vessels which indicate hemorrhage (R), infiltration of tissue by inflammatory cells (I), Occlusion of blood vessels with hyaline material (O), Figure S2: The photomicrographs of Fusidic Acid gel treated group show high improvement except small area of not complete re-epithelialization, Figure S3: The photomicrographs of Test (CRB-NE1 loaded chitosan gel) group show high improvement and almost normal tissue, Figure S4: The photomicrographs of control group show decrease in amount of collagen fibers indicated by decrease in blue color, Figure S5: The photomicrographs of Fusidic Acid gel treated group show high improvement by retaining amount and distribution of collagen fibers, Figure S6: The photomicrographs of Test (CRB-NE1 loaded chitosan gel) group show high improvement in amount and distribution of collagen fibers (blue color), Figure S7: The photomicrographs of control group show decrease of skin elasticity due to decrease in the amount of elastic fibers indicated by decrease in distribution of black color of Verhoef hematoxylin stain, Figure S8: The photomicrographs of Fusidic Acid gel treated group show high improvement and retaining elasticity of the skin, Figure S9: The photomicrographs of Test (CRB-NE1 loaded chitosan gel) group show high improvement and retaining elasticity of the skin.
