# Supplementary material for: Crisaborole Loaded Nanoemulsion Based Chitosan Gel: Formulation, Physicochemical Characterization and Wound Healing Studies
Source: Gels. 2022 May 19;8(5):318. doi: 10.3390/gels8050318 (PMC9140491; doi:10.3390/gels8050318)
Supplement: Supplementary file 1 [file gels-08-00318-s001.zip › gels-1704148-supplementary.pdf]

H&E stain

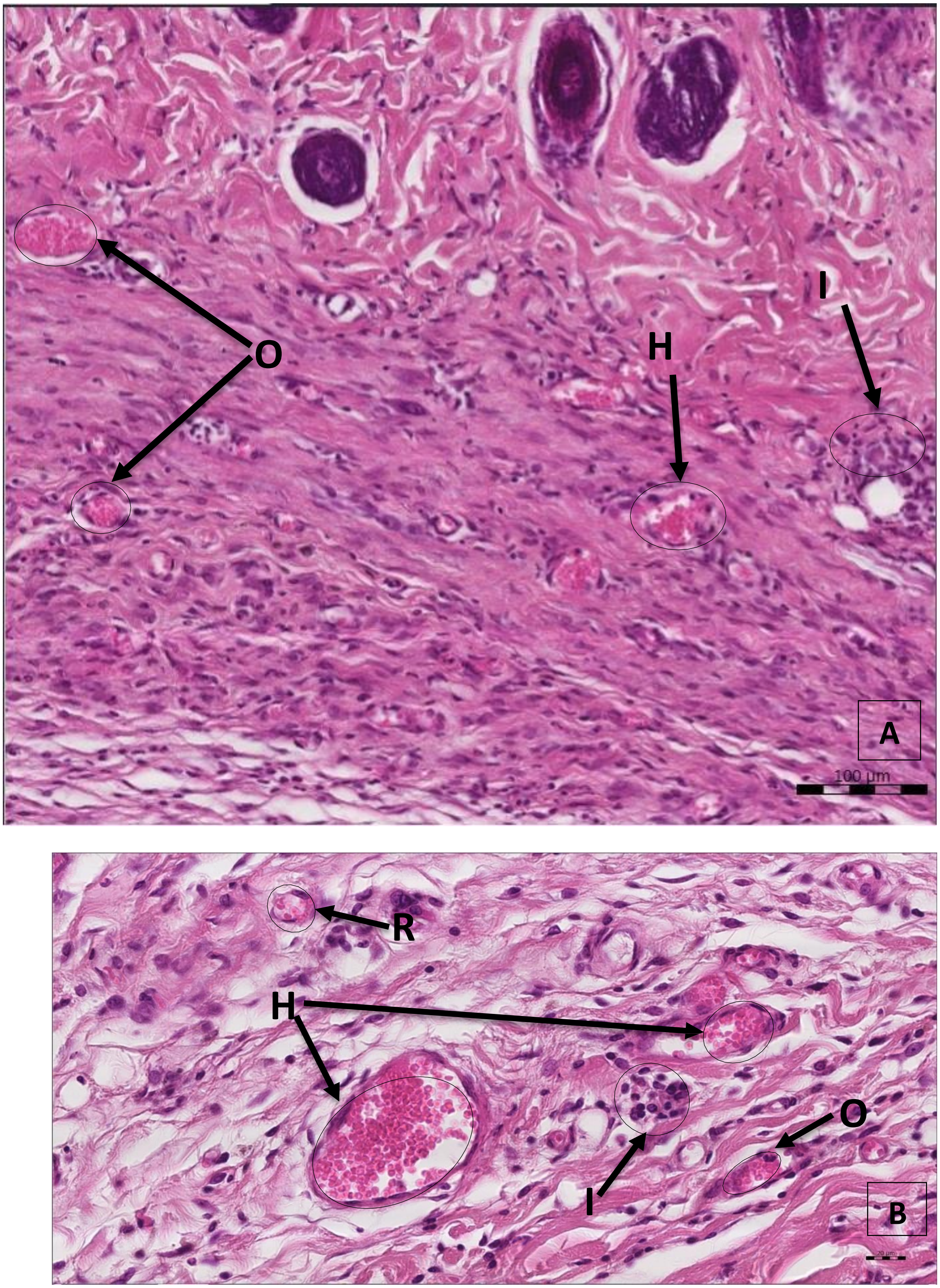

Figure S1: These photomicrographs of control group indicate suffering of skin tissue at both levels of dermis and epidermis indicated by several pathological events such as Hyperemia (H), presence of red blood cells within tissue outside blood vessels which indicate hemorrhage (R), infiltration of tissue by inflammatory cells (I), Occlusion of blood vessels with hyaline material (O). Photomicrograph A (Magnification 100x, scale bar 100 µm); photomicrograph B (Magnification 400x, scale bar 20 µm).

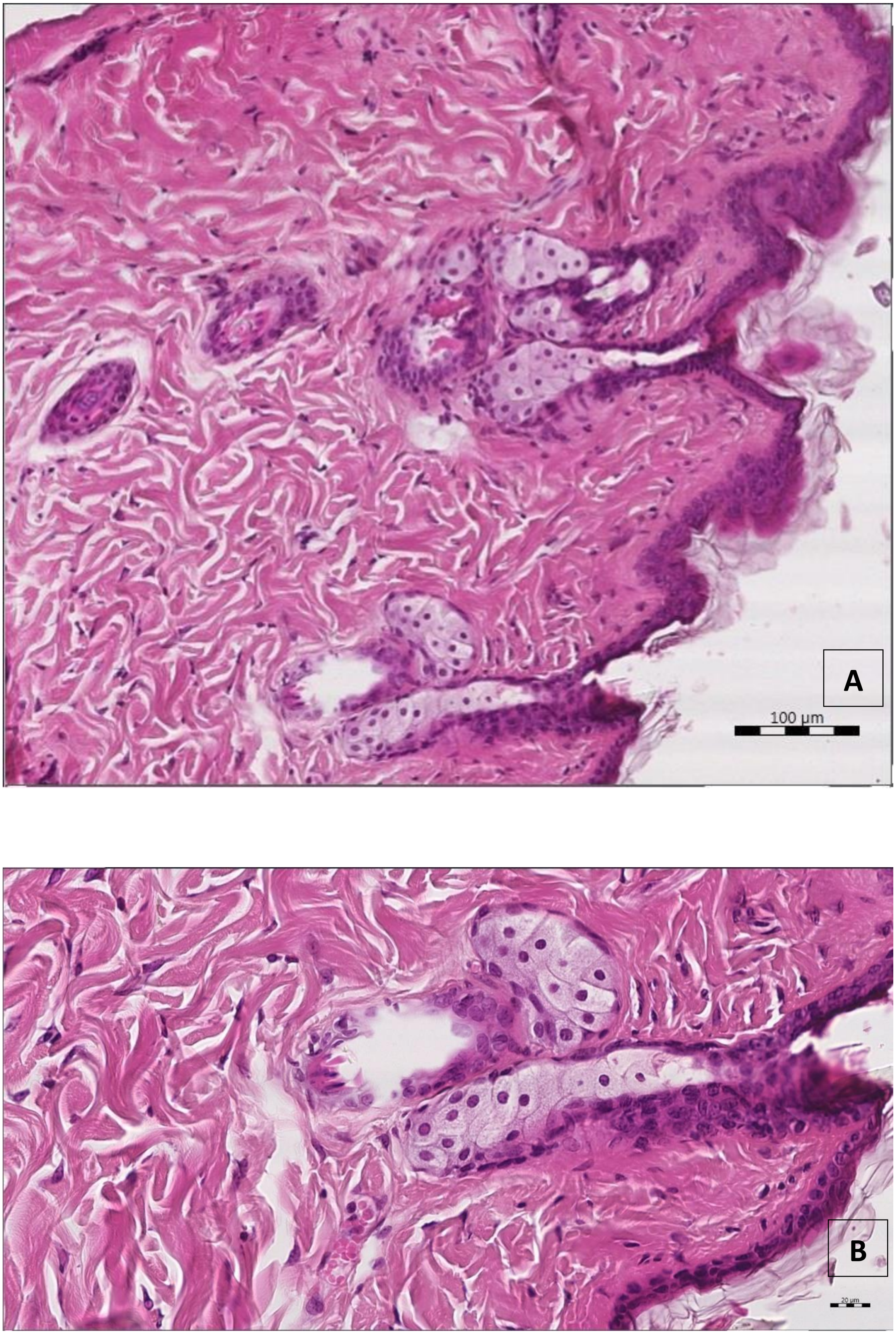

Figure S2: The photomicrographs of Fusidic Acid gel treated group show high improvement except small area of not complete re-epithelialization. Photomicrograph A (Magnification 100x, scale bar 100 μm); photomicrograph B (Magnification 400x, scale bar 20 μm).

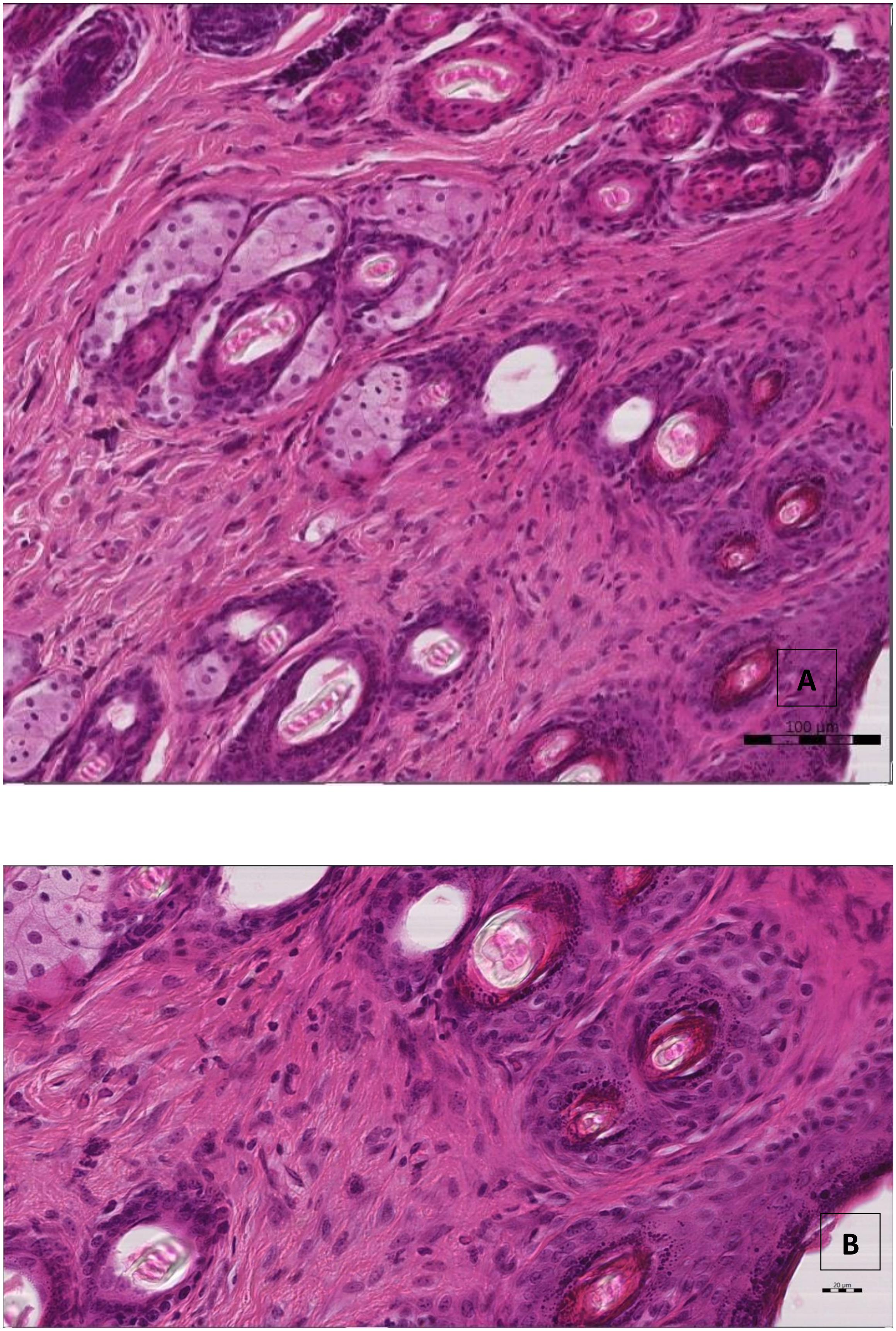

Figure S3: The photomicrographs of Test (CRB-NE1 loaded chitosan gel) group show high improvement and almost normal tissue. Photomicrograph A (Magnification 100x, scale bar 100 μm); photomicrograph B (Magnification 400x, scale bar 20 μm).

Masson Trichrome (MT) stain

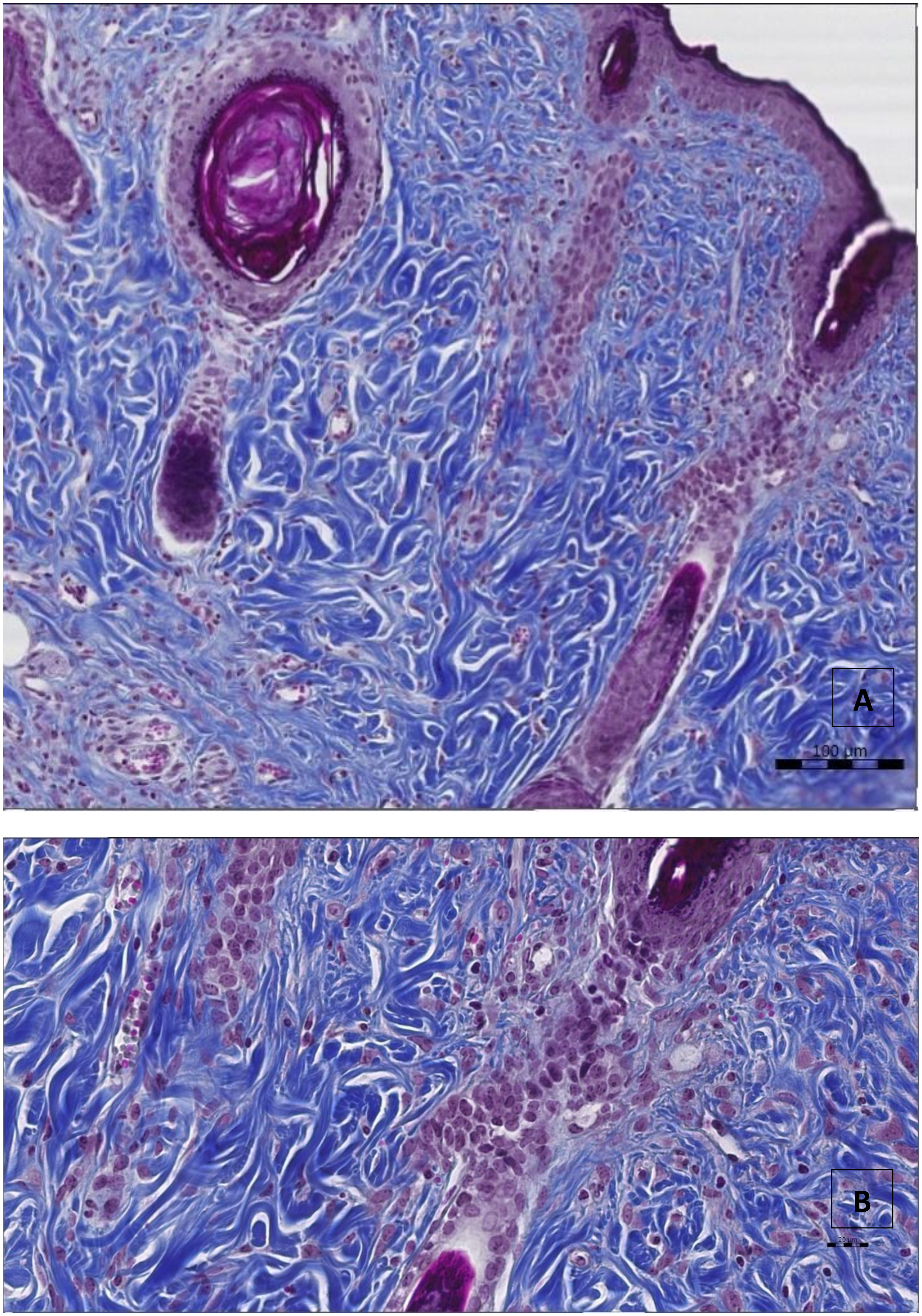

Figure S4: The photomicrographs of control group show decrease in amount of collagen fibers indicated by decrease in blue color. Stain is Masson trichrome for connective tissue fibers. Photomicrograph A (Magnification 100x, scale bar 100 µm); photomicrograph B (Magnification 400x, scale bar 20 µm).

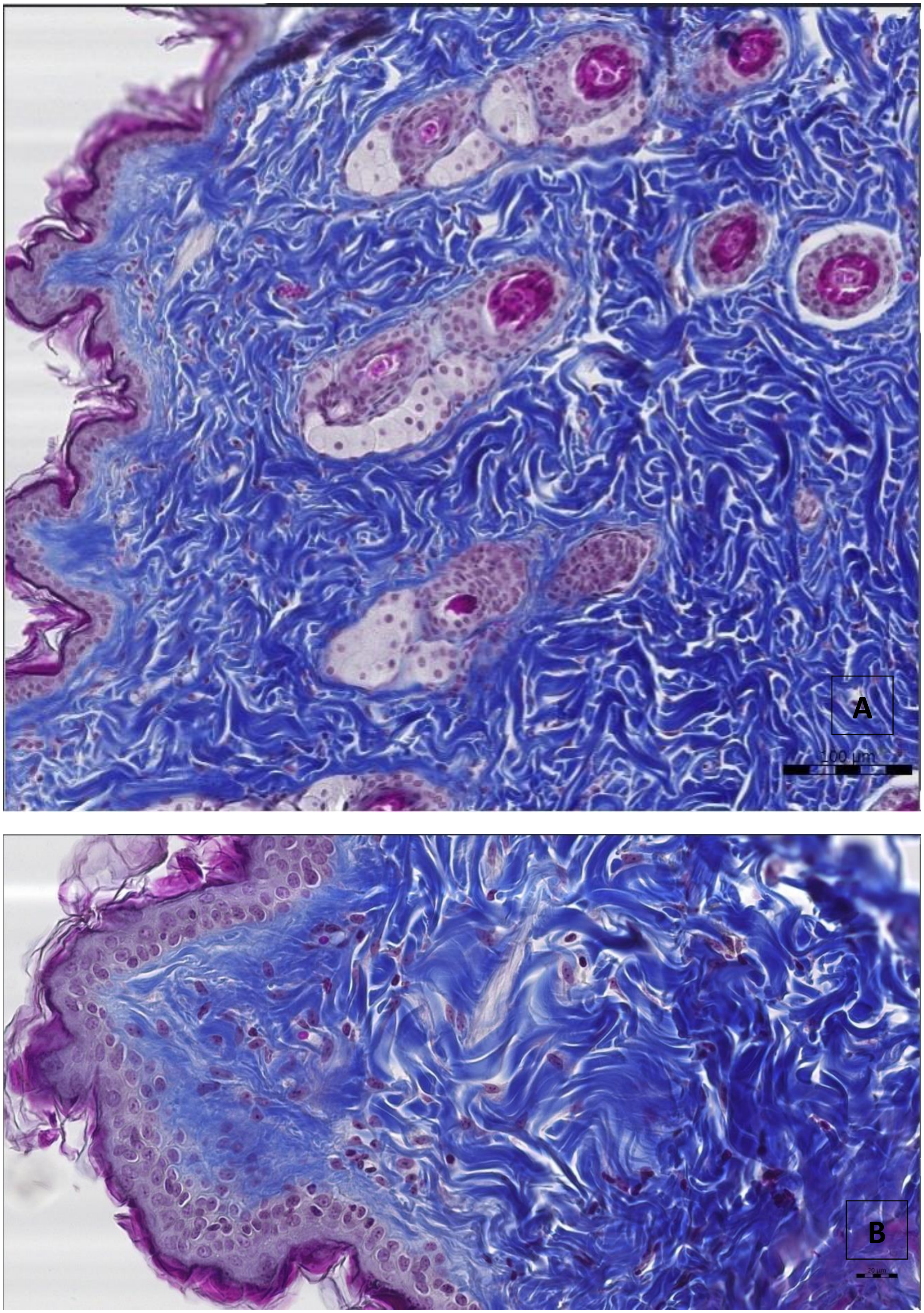

Figure S5: The photomicrographs of Fusidic Acid gel treated group show high improvement by retaining amount and distribution of collagen fibers. Stain is Masson trichrome for connective tissue fibers. Photomicrograph A (Magnification 100x, scale bar 100 µm); photomicrograph B (Magnification 400x, scale bar 20 µm).

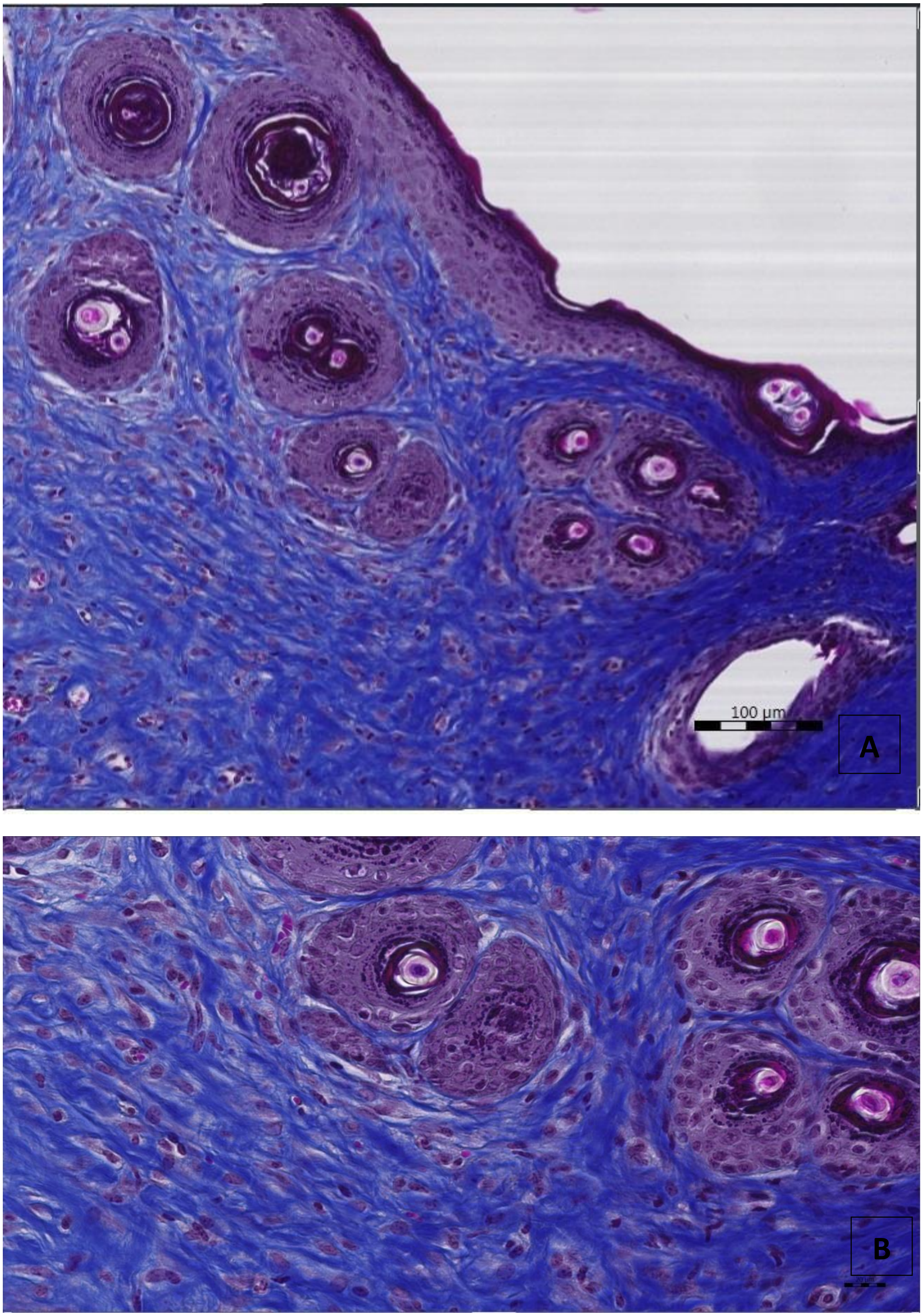

Figure S6: The photomicrographs of Test (CRB-NE1 loaded chitosan gel) group show high improvement in amount and distribution of collagen fibers (blue color). Stain is Masson trichrome for connective tissue fibers. Photomicrograph A (Magnification 100x, scale bar 100 µm); photomicrograph B (Magnification 400x, scale bar 20 µm).

Verhoef/vanGeison stain

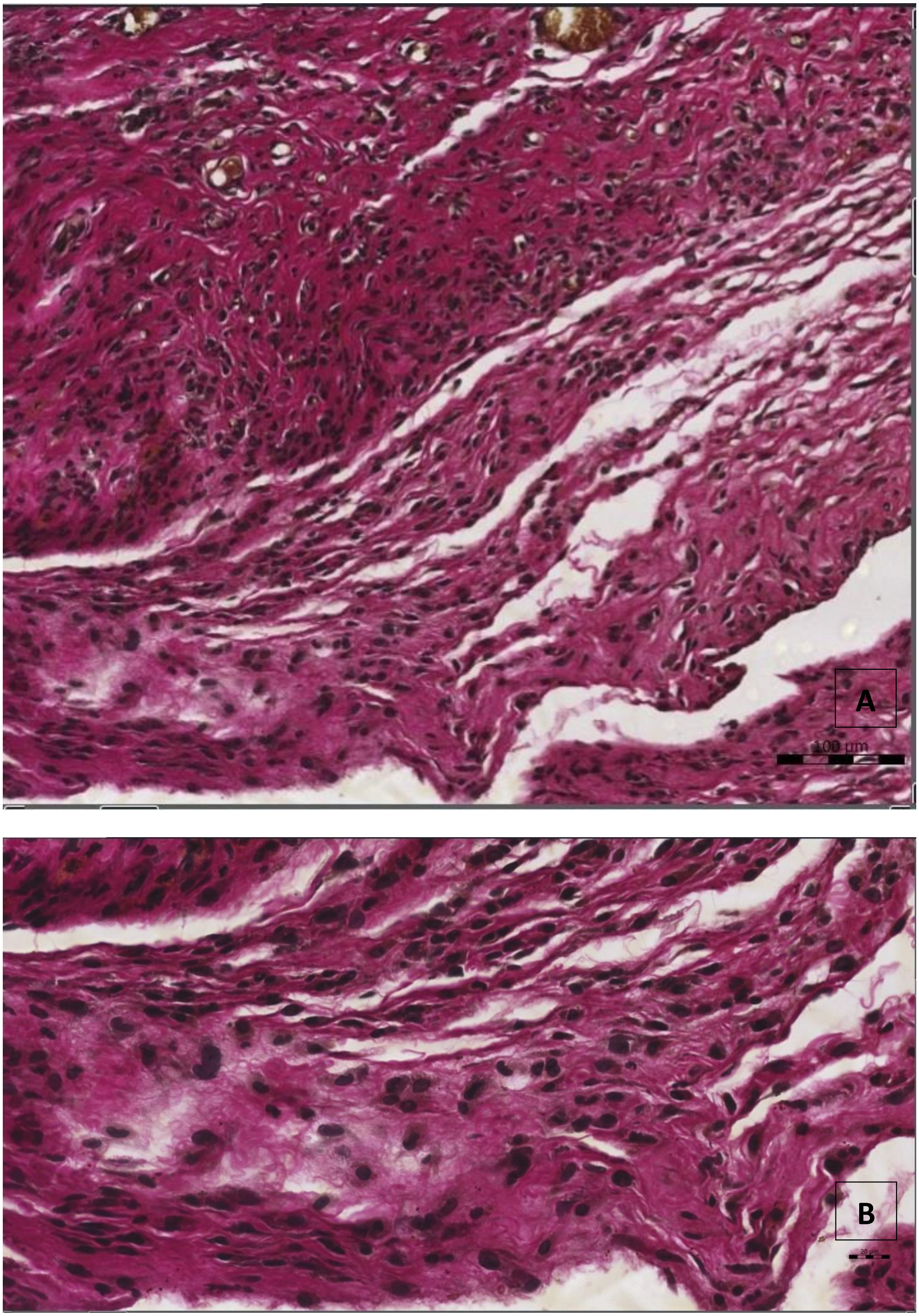

Figure S7: The photomicrographs of control group show decrease of skin elasticity due to decrease in the amount of elastic fibers indicated by decrease in distribution of black color of Verhoef hematoxylin stain. Stain is Verhoef for elastic fibers. Photomicrograph A (Magnification 100x, scale bar 100 μm); photomicrograph B (Magnification 400x, scale bar 20 μm).

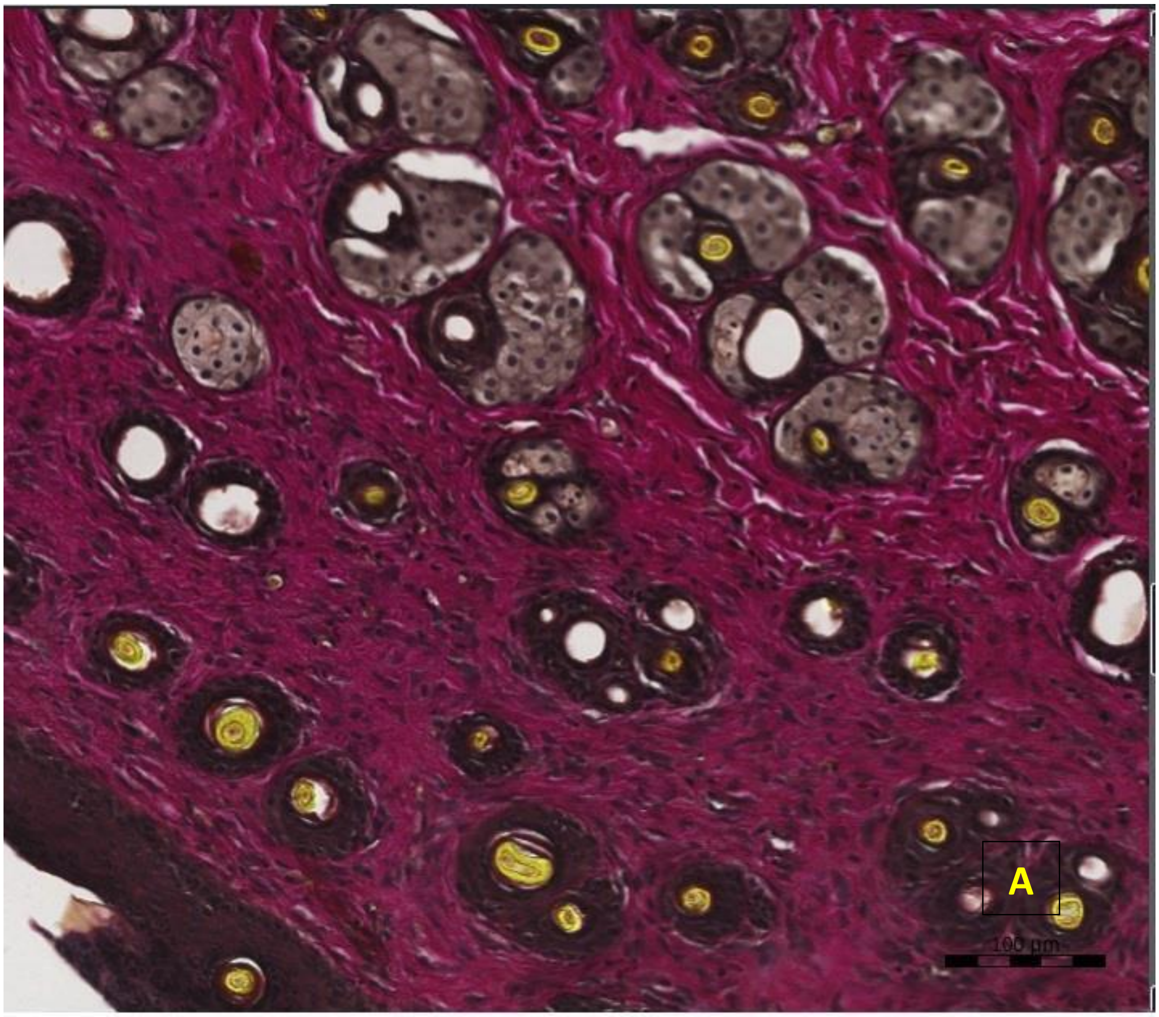

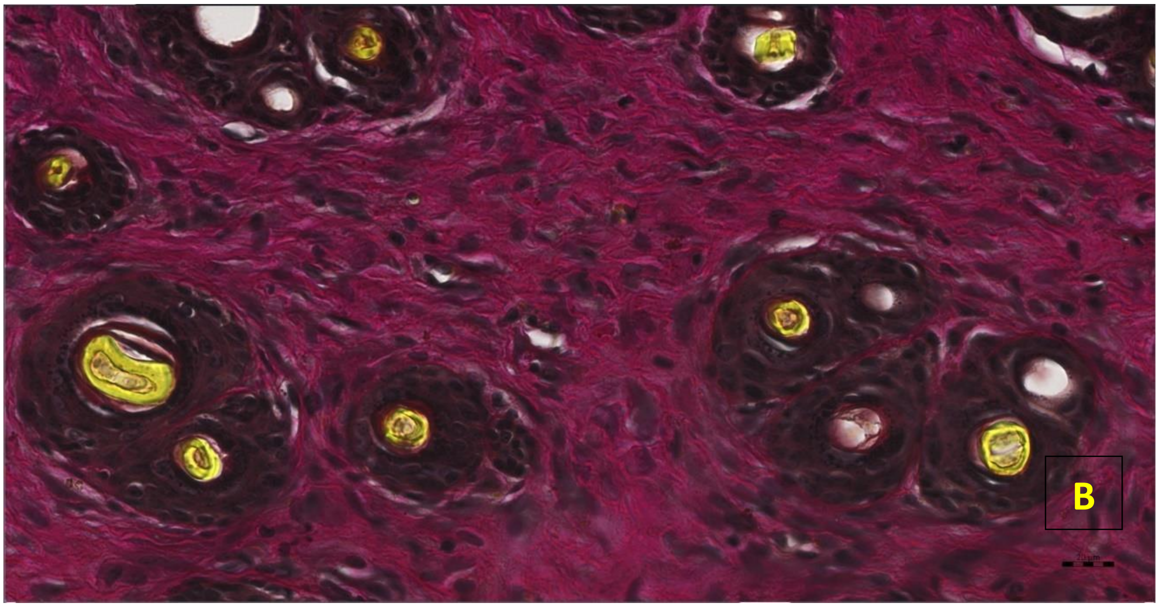

Figure S8: The photomicrographs of Fusidic Acid gel treated group show high improvement and retaining elasticity of the skin. Stain is Verhoef stain for elastic fibers. Photomicrograph A (Magnification 100x, scale bar 100 μm); photomicrograph B (Magnification 400x, scale bar 20 μm).

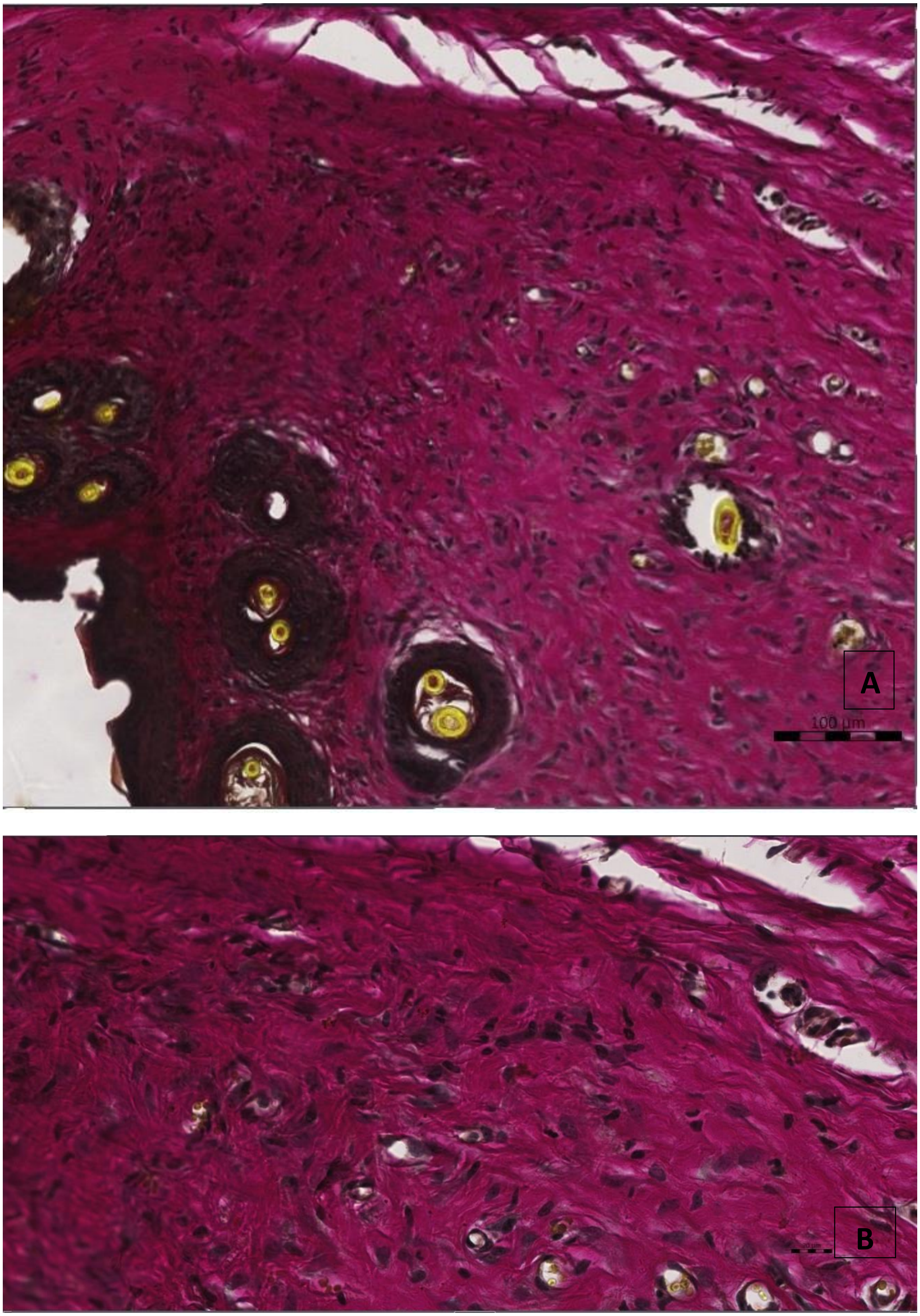

Figure S9: The photomicrographs of Test (CRB-NE1 loaded chitosan gel) group show high improvement and retaining elasticity of the skin. Stain is Verhoef stain for elastic fibers. Photomicrograph A (Magnification 100x, scale bar 100 µm); photomicrograph B (Magnification 400x, scale bar 20 µm).
